# Thermal Decomposition and Nonisothermal Kinetics of Monoethanolamine Mixed with Various Metal Ions

**DOI:** 10.1038/s41598-018-38434-1

**Published:** 2019-02-07

**Authors:** Mengning Wei, An-Chi Huang, Chi-Min Shu, Lijing Zhang

**Affiliations:** 1 College of Safety Science and Engineering, Nanjing Tech University, Nanjing, 210009 Jiangsu P. R. China; 2 Graduate School of Engineering Science and Technology, National Yunlin University of Science and Technology (YunTech), Yunlin, 64002 Taiwan Republic of China; 3 Department of Safety, Health, and Environmental Engineering, YunTech, Yunlin, Taiwan 64002 Republic of China

## Abstract

Ethanolamine is a critical chemical for petrochemical enterprises. When corrosion occurs in pipelines, equipment, and containers in petrochemical enterprises, minute amounts of metal ions are released. In this study, the thermal decomposition and nonisothermal kinetics of monoethanolamine (MEA) and MEA mixed with copper and zinc ions were analyzed using thermogravimetry (TG) and differential scanning calorimetry (DSC). The TG tests revealed that MEA mixed with copper (II) and zinc (II) began thermal decomposition at 75.2 and 60.3 °C, respectively, whereas pure MEA began thermal decomposition at 89.7 °C. Two exothermic peaks were observed in the DSC curves for MEA mixed with copper (II) and zinc (II), and thermokinetic parameters were obtained from DSC data. The apparent activation energy (*E*_a_) of each stage was calculated using several nonisothermal kinetic methods, namely the ASTM E698, Kissinger–Akahira–Sunose, Starink, and Flynn–Wall–Ozawa methods. The *E*_a_ of pure MEA was 28.7 ± 2.5 kJ/mol, whereas that of the copper and zinc mixtures were 80.5 ± 1.1 and 46.8 ±1.7 kJ/mol, respectively. The results can be used to improve the intrinsic safety of storage tanks and petrochemical plants.

## Introduction

As a derivative of ethylene oxide, ethanolamine is a valuable product of amino alcohol consisting of monoethanolamine (MEA), diethanolamine (DEA), and triethanolamine (TEA). Ethanolamine may be used for a variety of applications, including use in the intermediates of pesticides^1^, medicines^2^, detergent emulsifiers, resins^3^, and rubber^4^. Ethanolamine may also be used in desulfurization processes to remove acid gas and in the decarburization designs of refineries^5^. Ethanolamine is colorless, viscous, volatile, unstable, easily oxidized, corrosive^6^, and may cause fire or explosions when exposed to potent oxidants, such as hydrogen peroxide in a heated container^7^. Thermal decomposition and combustion may occur during the preparation, transportation, or storage of ethanolamine because of temperature imbalance and heat accumulation. At a company in Taoyuan, Taiwan in 2007, a fire and explosion occurred in a storage tank area containing 17 tanks of chemicals. The raw chemical materials involved in the accident primarily comprised glacial acetic acid, propylene glycol methyl ether, diethylene glycol butyl ether, and MEA.

The petrochemical sector has begun to expand, and petrochemical facilities are connected by a multitude of pipelines. Common metal pipeline materials include copper, zinc, and iron^8^. Pipeline corrosion is a critical concern for petrochemical enterprises; long-term corrosion of pipelines may lead to leaks and ruptures. In such cases, fire or explosions will occur if a transported material is inflammable or explosive and encounters an effective ignition source. Additionally, pipeline corrosion releases metal ions with free radicals^9^, which causes catalytic decomposition reactions that play a critical role in chain reactions and result in exothermic events. Corroded pipelines have caused leakages, fires, and explosion accidents worldwide, resulting in considerable economic losses, negative social aftermath, and environmental hazards. The Kaohsiung gas explosion and Chevron refinery fire incident were caused by corroded pipelines and led to extensive economic, social, and environmental damages^10^.

Generally, if corroded pipelines ethanolamine, the content of metal ions in the ethanolamine will increase gradually; the excess metal content then causes ethanolamine to foam and become unusable as a cleaning gas. Thus, the metal content of ethanolamine directly affects its quality and cost. More critically, this metal content provokes an incompatibility reaction, which induces advanced catalyzation of the material; consequently, the subsequent reaction cannot be controlled. Ávila^6^ investigated the thermal decomposition of MEA, DEA, TEA, and methyldiethanolamine (MDEA) and concluded that MEA exhibited the lowest thermal stability. In other studies, scholars^11,12^ have examined ethanolamine complexities using numerous methods, such as mixing ethanolamine with various oxidation states of vanadium. However, the thermal behavior of ethanolamine combined with metal ions has not been addressed in the literature; hence, the mechanism of metal ions in the thermal decomposition of ethanolamine should be determined.

The present study observed changes in the caloric value of MEA with the addition of various metal ions, namely copper (II) and zinc (II), by using thermogravimetry (TG), differential scanning calorimetry (DSC), and thermokinetic parameters, such as heat of decomposition (Δ*H*_d_), exothermic onset temperature (*T*_0_), and peak temperature (*T*_p_)^13–15^, were obtained. The results revealed that the decomposition of MEA mixed with copper (II) and zinc (II) exhibited a reaction earlier than did the pure MEA. The apparent activation energy (*E*_a_) of each stage was calculated using various thermokinetic models. The *E*_a_ of pure MEA was 28.7 ± 2.5 kJ/mol, whereas that of the copper and zinc mixtures were 80.5 ± 1.1 and 46.8 ± 1.7 kJ/mol, respectively. The results of this study may serve as a reference for the preparation, application, usage, storage, and disposal of MEA, and could be used to minimize thermal risk and enhance the intrinsic safety of storage tanks in petrochemical plants.

## Experimental and Methods

### Sample preparations

MEA of 99 mass% purity was purchased from Acros Organics (Thermo Fisher Scientific Ltd., New Jersey, USA). The chemical formula of MEA is C_2_H_7_NO, and its Chemical Abstracts Service number is 141-43-5. Copper and zinc ions, which are common metallic materials used in pipelines, were separated from CuBr_2_ and ZnBr_2_ (provided by Alfa Aesar Ltd., Haverhill, MA, USA). To prevent deterioration, all samples were stored in a dry and dark place.

### TG experiments

TG experiments were performed using a Perkin Elmer Pyris 1 thermogravimetric analyzer (Waltham, Massachusetts, USA) with a balanced furnace and vertical design. TG and differential TG (DTG) curves revealed variations in the mass loss and its derivative when the temperature was increased^16,17^. For pure MEA samples, the experimental temperature ranged from 30.0 to 300.0 °C and heating rates of 5.0, 10.0, 15.0, 20.0, and 25.0 °C/min were applied. In total, 10.0 mg of samples were used. Experiments using MEA mixed with CuBr_2_ and ZnBr_2_ were conducted at a heating rate of 10.0 °C/min within the temperature range of 30.0–650.0 °C. All samples were placed in a platinum crucible. Tests proceeded under an air atmosphere with 20.0 mL/min flow. Three sets of experiment were performed to ensure the reliability of the results and experimental methodology.

### DSC experiments

Thermal analysis tests were performed using a Mettler Toledo DSC-821 (Mettler Toledo International Inc., Columbus, OH, USA). Because of its ease and efficiency of operation, DSC is the standard instrument used in conventional thermoanalysis^18^. A DSC analysis was conducted to generate thermal curves denoting the temperature ranges of exothermic and endothermic reactions. Heat-flow data could be obtained from the area of the reactions^19^. The DSC-821 has been calibrated of heating rate at 4.0 °C/min before the experiment. In this study, DSC experiments were performed at heating rates of 2.0, 4.0, 6.0, 8.0, and 10.0 °C/min, and the test temperature range was set at 30.0–400.0 °C. STAR^e^ software was used to establish thermokinetic models and obtain kinetic parameters^20,21^. The sample sizes of MEA, MEA mixed with CuBr_2_, and MEA mixed with ZnBr_2_ were approximately 7.0 mg in each experiment. The sample ratio was approximately 2:1.

### Nonisothermal kinetic methods

The dynamic parameter *E*_a_ was calculated in the nonisothermal experiment, which was performed in a growing environment. Kinetic analysis generally involves model-free and model-fitting methods^22^. The model-free method, also termed the isoconversional method, enhances analysis accuracy by excluding kinetic model functions^23–25^. In this method, the reaction rate is assumed to be only a function of temperature, and *E*_a_ values are compared at different conversions to corroborate the consistency of the reaction mechanism throughout the process^26^. Kinetic methods are divided into differential and integral methods^27^. In this study, the ASTM E698, Kissinger–Akahira–Sunose (KAS), Starink, and Flynn–Wall–Ozawa (FWO) methods were selected as representative methods for dynamic analysis. Table 1 presents the nonisothermal kinetic methods used for the present study and obtained from TG–DTG and DSC analysis to estimate the *E*_a_. All of these methods were transformed using the nonisothermal kinetic equation, as presented in Eq. (1):

1 $$\frac{{\rm{d}}\alpha }{{\rm{d}}T}=\frac{A}{\beta }\,\exp (-\frac{{E}_{{\rm{a}}}}{RT})f(\alpha )$$

**Table 1:** Kinetic methods used in this study.

Method	Expression	Plot	References
ASTM E698	$\mathrm{ln}(\frac{\beta }{{T}_{{\rm{p}}}^{2}})=Const-\frac{{E}_{{\rm{a}}}}{R{T}_{{\rm{p}}}}$	$\mathrm{ln}(\frac{\beta }{{T}_{{\rm{p}}}^{2}})$ against $\frac{1}{{T}_{{\rm{p}}}}$	^33^
KAS	$\mathrm{ln}(\frac{\beta }{{T}^{2}})=\,\mathrm{ln}(\frac{AR}{{E}_{{\rm{a}}}g(\alpha )})-\frac{{E}_{{\rm{a}}}}{RT}$	$\mathrm{ln}(\frac{\beta }{{T}^{2}})$ against $\frac{1}{T}$	^34, 35^
Starink	$\mathrm{ln}(\frac{\beta }{{T}^{{\rm{1.8}}}})={C}_{s}-1.0037\frac{{E}_{{\rm{a}}}}{RT}$	$\mathrm{ln}(\frac{\beta }{{T}^{{\rm{1.8}}}})$ against $\frac{1}{T}$	^36^
FWO	$\mathrm{log}\,\beta =\,\mathrm{ln}(\frac{A{E}_{{\rm{a}}}}{Rf(\alpha )})-2.315-0.4567\frac{{E}_{{\rm{a}}}}{RT}$	log*β* against $\frac{1}{T}$	^37^

The value of *E*_a_ was calculated with the slope of a line respectively by using the equations of these four methods. In terms of Starink method, ln (*β*/*T*^1.8^) was plotted against 1/*T* with a slope of −1.0037 *E*_a_/*RT* to obtain *E*_a_.

## Results and Discussion

### Thermogravimetric analysis through TG testing

**Figure 1:**
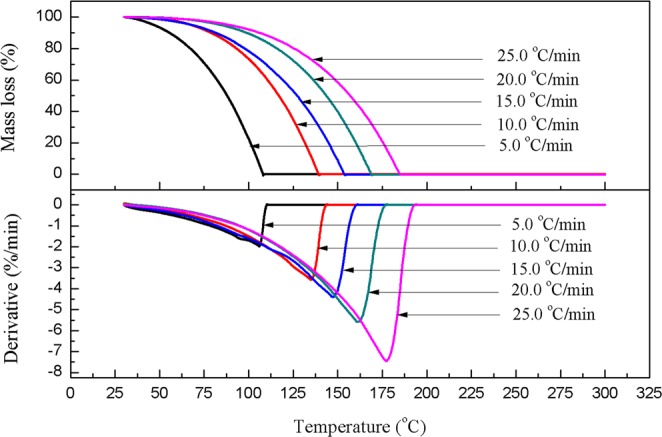
TG and DTG curves of MEA at heating rates of 5.0, 10.0, 15.0, 20.0, and 25.0 °C/min in an air atmosphere.

Figure 1 illustrates the TG and DTG curves of MEA at 5.0, 10.0, 15.0, 20.0, and 25.0 °C/min heating rates in an air atmosphere. The TG curves revealed that a single stage mass loss was observed within the temperature range of 50.0–200.0 °C, and decomposition was initially quick compared with other substances because of the volatility of MEA. The TG and DTG curves exhibited sufficient consistency. By increasing the heating rate, the reaction of mass loss became more intense; furthermore, the initial decomposition temperature (*T*_i_), maximum decomposition temperature (*T*_m_), and final decomposition temperature (*T*_f_) all increased, as did the rate of mass loss, which reached 7.4%/min at a heating rate of 25.0 °C/min. The decomposition of all heating rates was complete without residues before the temperature reached 200.0 °C.

**Figure 2:**
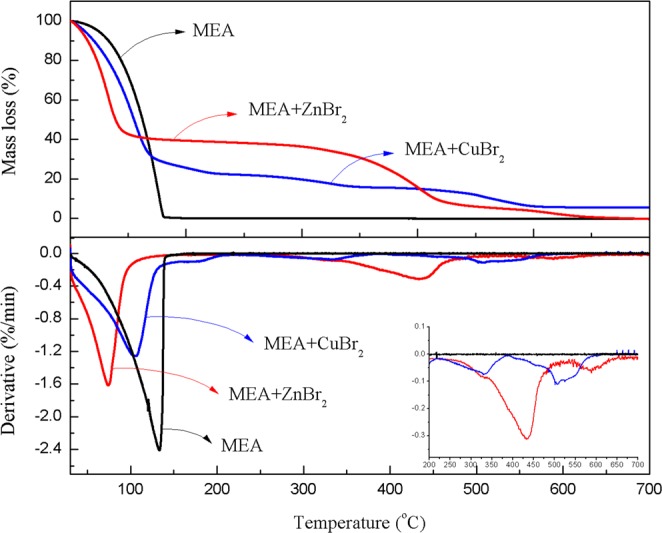
TG and DTG curves of MEA, MEA mixed with CuBr_2_, and MEA mixed with ZnBr_2_ at a heating rate of 10.0 °C/min in an air atmosphere.

The TG and DTG curves of MEA and MEA individually mixed with CuBr_2_ and ZnBr_2_ at a heating rate of 10.0 °C/min in an air atmosphere are presented in Fig. 2. As indicated in the diagram, the mass-loss process of MEA mixed with CuBr_2_ consists of three stages. The first stage, which occurred between 50.0 and 230.0 °C with a mass loss of 78.3%, was attributed to the reaction of MEA and CuBr_2_. The second stage, which occurred between 230.0 and 450.0 °C with a mass loss of 6.9%, represented the decomposition of the remaining CuBr_2_. The third stage, which occurred between 450.0 and 700.0 °C, resulted in the formation of 5.6% of residues and corresponded to the formation of CuO. For MEA mixed with ZnBr_2_, the TG and DTG curves indicated two stages of mass loss. The first decomposition (30.0–200.0 °C) resulted in 60.0% mass loss and was caused by the reaction of MEA and ZnBr_2_. The mass loss (40.0%) of the second decomposition (at 200.0–700.0 °C) was caused by the decomposition of the remaining ZnBr_2_ without residues’ formation.

The calculated values of TG and DTG for various stages of the three samples are presented in Table 2, including the characteristic temperature and mass loss of each stage. As evident in these data, the reaction rate of MEA increased after copper (II) and zinc (II) had been added, and *T*_i_ and *T*_m_ decreased in the first stage. As indicated in Table 2, the addition of zinc (II) (*T*_i_ = 60.3 °C) catalyzed the reaction of MEA more quickly than did the addition of copper (II) (*T*_i_ = 75.2 °C).

**Table 2:** TG–DTG analysis results of MEA mixed with CuBr_2_ and MEA mixed with ZnBr_2_.

Samples	Stage	Temperature range (°C)	*T*_i_ (°C)	*T*_m_ (°C)	*T*_f_ (°C)	*W* (%)
MEA	I	30.0–200.0	89.7	133.0	148.3	100.0
MEA + CuBr_2_	I	30.0–220.0	75.2	103.0	229.2	78.3
	II	220.0–450.0	290.3	333.0	392.5	6.9
	III	450.0–650.0	506.6	506.6	599.3	9.2
MEA + ZnBr_2_	I	30.0–200.0	60.3	74.0	138.0	60.0
	II	200.0–700.0	360.9	433.3	667.0	40.0

### Thermodynamics of DSC tests

**Figure 3:**
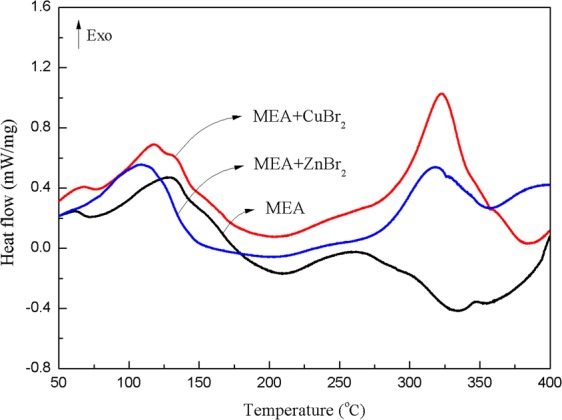
DSC curves for MEA, MEA mixed with CuBr_2_, and MEA mixed with ZnBr_2_ at a heating rate of 8.0 °C/min.

The DSC curves of the three samples at a heating rate of 8.0 °C/min are displayed in Fig. 3. For MEA, exothermic and endothermic peaks were observed at 120.0 and 320.0 °C, respectively. The exothermic onset temperature (*T*_0_) and maximum decomposition temperature (*T*_p_) were 89.5 and 130.3 °C, respectively. *T*_0_ can be defined by the intersection of a line drawn tangent to the steepest slope of the curve with the baseline; moreover, *T*_p_ is the maximum exothermic temperature that can be achieved in the exothermic interval. The curves of MEA mixed with CuBr_2_ and MEA mixed with ZnBr_2_ produced two exothermic peaks at approximately 120.0 and 320.0 °C, respectively. The first peak indicated that advanced canalization occurred after the addition of CuBr_2_ and ZnBr_2_ to MEA. *T*_0_ decreased to 79.2 and 69.6 °C, respectively, which is consistent with the results of the TG test, and *T*_p_ decreased to 117.6 and 110.5 °C, respectively. Δ*H*_d_ was similar in the first peak for all three samples, namely 185.4, 199.6, and 151.6 J/g, respectively. For the second exothermic peak of MEA mixed with CuBr_2_ and ZnBr_2_, the respective values of *T*_p_ were 322.8 and 316.7 °C. The peak may be considered representative of the thermal decomposition of the remaining bromide. In this test, the influence of Br^−^ in the decomposition of MEA could be disregarded because both metallic compounds contained negative ions.

**Figure 4:**
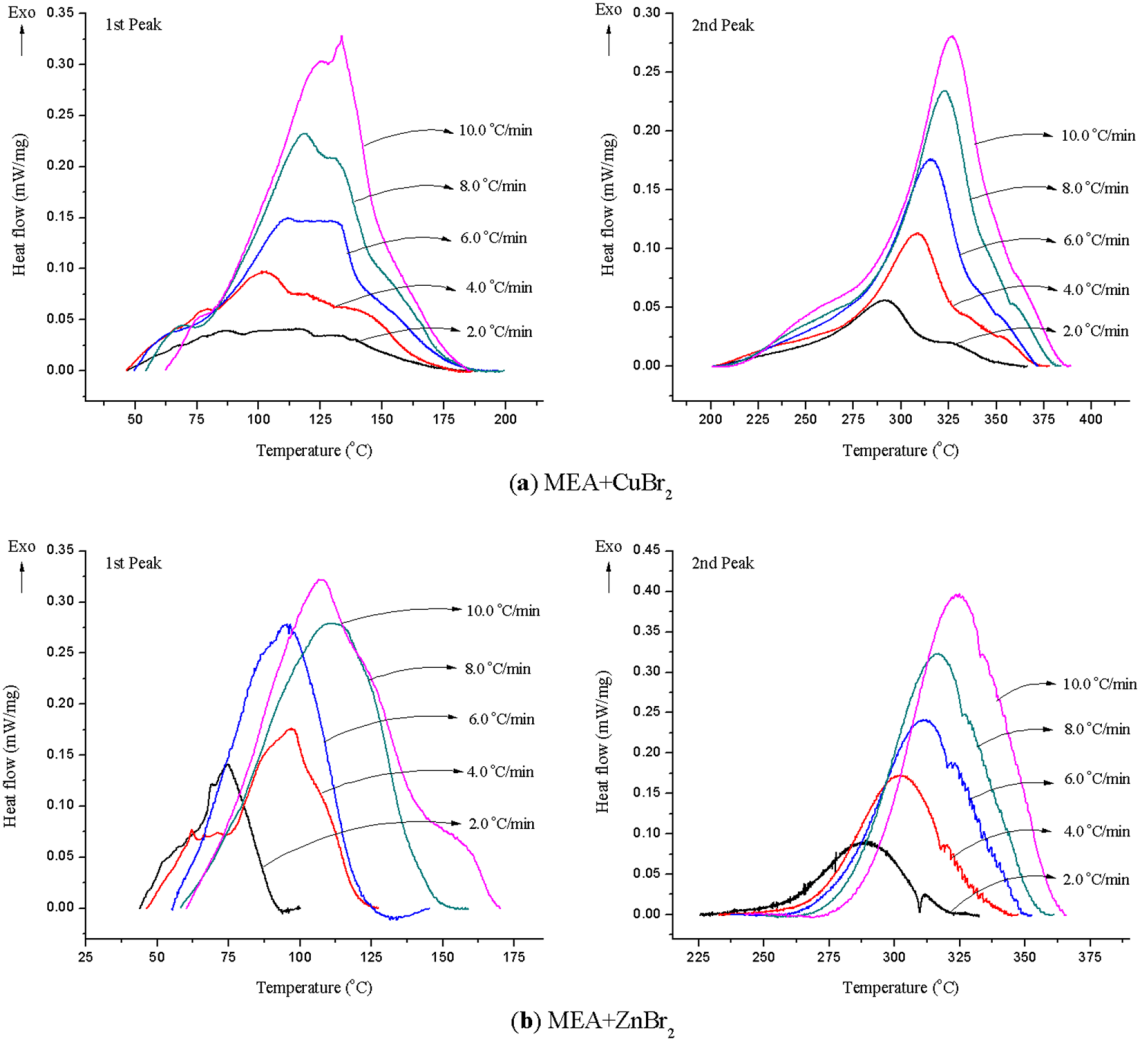
DSC curves for MEA mixed with (**a**) CuBr_2_ and (**b**) ZnBr_2_ at heating rates of 2.0, 4.0, 6.0, 8.0, and 10.0 °C/min.

Figure 4 displays the temperature–heat flow curves of MEA mixed with (a) CuBr_2_ and (b) ZnBr_2_ at heating rates of 2.0, 4.0, 6.0, 8.0, and 10.0 °C/min. The DSC curves exhibited similar trends. The exothermic intervals of the first and second peaks were 50.0–180.0 and 200.0–380.0 °C, respectively, and *T*_0_ and *T*_p_ were delayed as the heating rates increased. For MEA mixed with ZnBr_2_ in the DSC experiments, regularity among *T*_01_, *T*_p1_, and Δ*H*_d1_ with the increasing heating rates in the first peak could not be confirmed, as indicated in Fig. 4(b). These results can be attributed to the water adsorption of ZnBr_2_, which rendered the initial reaction unstable. However, the values of Δ*H*_d_ in the second peak at different heating rates were between 99.5 and 124.7 J/g. For MEA mixed with CuBr_2_, the Δ*H*_d_ results of the first and second peaks at five heating rates were 187.4–237.8 and 373.6–471.9 J/g, respectively. All thermokinetic parameter results are listed in Table 3.

**Table 3:** Thermodynamic data from the DSC curves of MEA mixed with CuBr_2_ and MEA mixed with ZnBr_2_ at heating rates of 2.0, 4.0, 6.0, 8.0, and 10.0 °C/min.

Samples	*β* (°C/min)	Mass (mg)	*T*_01_ (°C)	*T*_p1_ (°C)	Δ*H*_d1_ (J/g)	*T*_02_ (°C)	*T*_p2_ (°C)	Δ*H*_d2_ (J/g)
MEA + CuBr_2_	2.0	6.58	54.8	85.3	195.3	273.0	291.0	406.0
	4.0	6.85	70.2	101.2	187.4	278.0	308.6	471.9
	6.0	6.93	78.3	111.4	203.4	281.0	315.3	373.6
	8.0	6.87	79.2	117.6	199.6	285.1	322.1	419.2
	10.0	6.67	86.0	133.7	206.6	287.3	326.8	419.6
MEA + ZnBr_2_	2.0	7.30	64.7	74.5	78.2	263.0	289.0	99.5
	4.0	7.85	66.1	96.8	101.2	272.5	301.7	124.76
	6.0	7.41	68.6	96.5	117.2	280.6	311.9	113.16
	8.0	7.52	69.6	110.4	151.5	287/3	316.7	119.21
	10.0	7.21	71.5	106.5	97.1	292.5	324.7	113.78

### Calculations and comparison of *E*_a_

*E*_a_ is a crucial kinetic parameter for evaluating the reactability of a substance. To further explore the effects of adding copper (II) and zinc (II) on the thermal stability of MEA, various nonisothermal kinetic methods were used to analyze the exothermic peaks in the DSC tests. *E*_a_ results were calculated for MEA and MEA mixtures at each experimental stage.

**Figure 5:**
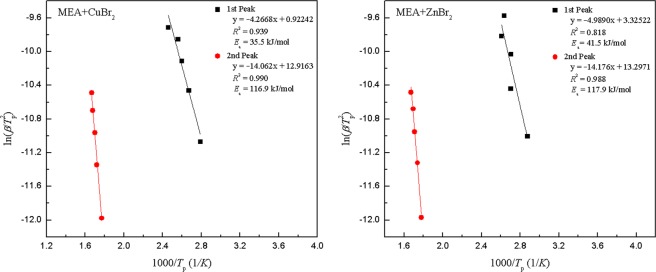
Plots of $\mathrm{ln}(\beta /{T}_{{\rm{p}}}^{2})$ versus 1/*T*_p_ for MEA mixed CuBr_2_ and MEA mixed with ZnBr_2_ for four exothermal peaks calculated using the ASTM E698 method.

The ASTM E698 was first used to calculate the *E*_a_ of each set of experiments. Figure 5 displays the plots of $\mathrm{ln}\,(\beta /{T}_{{\rm{p}}}^{2})$ versus 1/*T*_p_ for the four exothermic peaks according to the ASTM E698 method. The *E*_a_ results for the first and second peaks of MEA mixed with CuBr_2_ were 30.3 and 115.6 kJ/mol, and those for MEA mixed with ZnBr_2_ were 42.1 and 116.9 kJ/mol, respectively. However, the ASTM E698 method could not be used to reveal the overall trend of *E*_a_ at each conversion. The values for *E*_a_ acquired using the ASTM E698 method were slightly inaccurate for these experiments because of the basic assumptions of the method^28^. Subsequently, the KAS, Starink, and FWO methods were used for different conversions. The DSC curve exhibited artificiality in baseline selection during processing and analysis, especially for reactions at the beginning and end with device noise. Therefore, the conversion at the interval of *α* between 0.1 and 0.9 was selected for the kinetic analysis.

**Figure 6:**
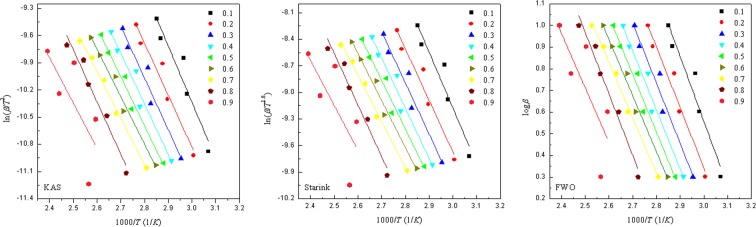
Plots of log*β* versus 1/*T* for the first peak of MEA mixed with ZnBr_2_ determined using the KAS, Starink, and FWO methods at different conversions.

**Figure 7:**
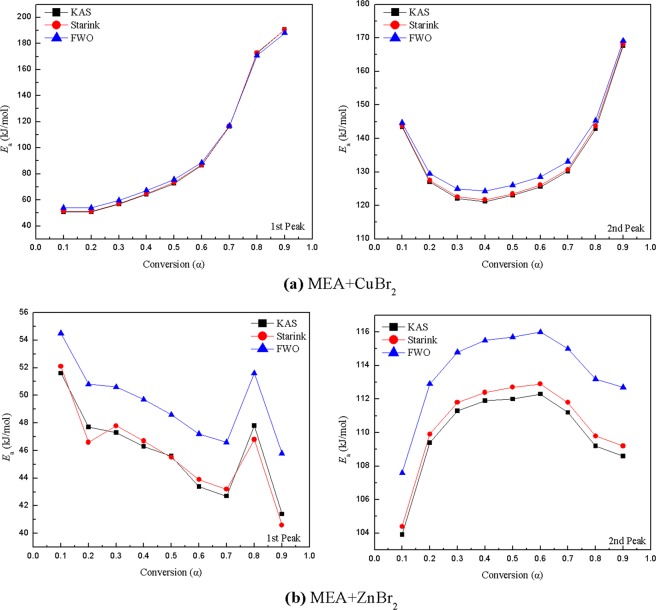
Variation of *E*_a_ at different conversions for (**a**) MEA mixed with CuBr_2_ and (**b**) MEA mixed with ZnBr_2_.

For the KAS, Starink, and FWO methods, general trends in *E*_a_ values can be identified at different conversions. Plots for the first peak of MEA mixed with ZnBr_2_ obtained using these three methods at different conversions are displayed in Fig. 6; the fitting plots deviated substantially at the conversions of 0.1–0.2 and 0.8–0.9. At the conversion of 0.3–0.7, the fitting plots were nearly parallel. Accordingly, the average of *E*_a_ was calculated within the interval of 0.3–0.7, as suggested in relevant studies^22,29^. The plots of other peaks that are not displayed in Fig. 6 were similar to this figure after fitting. *E*_a_ was readily calculated at different conversions using the slope of the curves in Fig. 6. Tables 4 and 5 list the *E*_a_ of MEA mixed with CuBr_2_ and ZnBr_2_ at different conversions. The variation of *E*_a_ at different conversions for these four exothermic peaks according to the KAS, Starink, and FWO methods is represented in Fig. 7. Similar results for *E*_a_ were obtained with the KAS and Starink methods, whereas the FWO method yielded a slightly higher *E*_a_ value.

**Table 4:** *E*_a_ for MEA mixed with CuBr_2_, calculated using the KAS, Starink, and FWO methods at different conversions.

Conversion (*α*)	1st Peak						2nd Peak					
	KAS		Starink		FWO		KAS		Starink		FWO	
	*E*_a_ (kJ/mol)	*R* ^2^	*E*_a_ (kJ/mol)	*R* ^2^	*E*_a_ (kJ/mol)	*R* ^2^	*E*_a_ (kJ/mol)	*R* ^2^	*E*_a_ (kJ/mol)	*R* ^2^	*E*_a_ (kJ/mol)	*R* ^2^
0.1	50.9	0.689	51.3	0.694	54.0	0.737	143.4	0.978	143.7	0.978	144.7	0.980
0.2	50.7	0.748	51.1	0.753	54.0	0.791	127.0	0.998	127.5	0.997	129.5	0.998
0.3	56.7	0.810	57.1	0.814	59.7	0.842	122.1	0.994	122.6	0.994	125.0	0.995
0.4	64.4	0.805	64.8	0.809	67.2	0.834	121.2	0.992	121.7	0.993	124.3	0.994
0.5	72.6	0.748	73.4	0.758	75.6	0.787	123.0	0.992	123.5	0.992	126.1	0.993
0.6	86.7	0.733	87.0	0.736	88.6	0.762	125.6	0.992	126.1	0.992	128.6	0.993
0.7	116.4	0.788	116.5	0.790	116.9	0.807	130.2	0.991	130.7	0.991	133.1	0.992
0.8	172.8	0.842	172.9	0.844	170.8	0.853	142.8	0.984	143.7	0.984	145.3	0.986
0.9	190.7	0.897	190.7	0.898	188.0	0.904	167.7	0.963	168.1	0.963	169.2	0.967
Mean	79.4	0.777	79.8	0.781	81.6	0.806	124.4	0.992	124.9	0.992	127.4	0.993
Standard deviation	21.0	0.031	20.9	0.030	20.1	0.030	3.2	0.001	3.2	0.001	3.2	0.001

**Table 5:** *E*_a_ for MEA mixed with ZnBr_2_, calculated using the KAS, Starink, and FWO methods at different conversions.

Conversion (*α*)	1st Peak						2nd Peak					
	KAS		Starink		FWO		KAS		Starink		FWO	
	*E*_a_ (kJ/mol)	*R* ^2^	*E*_a_ (kJ/mol)	*R* ^2^	*E*_a_ (kJ/mol)	*R* ^2^	*E*_a_ (kJ/mol)	*R* ^2^	*E*_a_ (kJ/mol)	*R* ^2^	*E*_a_ (kJ/mol)	*R* ^2^
0.1	51.6	0.903	52.1	0.905	54.5	0.920	103.9	0.995	104.4	0.995	107.6	0.996
0.2	47.7	0.939	46.6	0.941	50.8	0.951	109.4	0.994	109.9	0.994	112.9	0.995
0.3	47.3	0.936	47.8	0.937	50.6	0.949	111.3	0.993	111.8	0.993	114.8	0.994
0.4	46.3	0.948	46.7	0.950	49.7	0.959	111.9	0.994	112.4	0.994	115.5	0.995
0.5	45.6	0.971	45.5	0.965	48.6	0.972	112.0	0.993	112.7	0.994	115.7	0.995
0.6	43.4	0.978	43.9	0.979	47.2	0.983	112.3	0.994	112.9	0.994	116.0	0.995
0.7	42.7	0.989	43.2	0.989	46.6	0.992	111.2	0.994	111.8	0.994	115.0	0.995
0.8	47.8	0.955	46.8	0.956	51.6	0.965	109.2	0.995	109.8	0.995	113.2	0.995
0.9	41.4	0.358	40.6	0.369	45.8	0.456	108.6	0.996	109.2	0.996	112.7	0.997
Mean	45.1	0.964	45.4	0.964	48.5	0.971	111.7	0.994	112.3	0.994	115.4	0.995
Standard deviation	1.7	0.020	1.7	0.019	1.5	0.016	0.4	0.001	0.5	0.001	0.4	0.001

Table 6 summarizes the calculated results of *E*_a_ and *R*^2^ for each nonisothermal method. The *E*_a_ of pure MEA was 28.7 ± 2.5 kJ/mol, which is lower than the value indicated in related studies^6^. For MEA mixed with CuBr_2_, the *E*_a_ of the first peak was 80.5 ± 1.1 kJ/mol, suggesting that the *E*_a_ of the mixture was higher than that of pure MEA. The data listed in Table 6, along with the curves delineated in Fig. 7, demonstrate that the *E*_a_ increased with conversion; this was likely because of a chemical reaction and the formation of a copper-alkanolamine complex^30^. The calculated value of *E*_a_ in the second stage was 125.9 ± 1.5 kJ/mol, which was slightly higher than the value reported in a relevant study^31^. This value may be attributable to the decomposition of the remaining CuBr_2_. For MEA mixed with ZnBr_2_, the *E*_a_ values of the first and second peaks were 46.8 ± 1.7 and 113.6 ± 1.9 kJ/mol, respectively. As indicated by the *R*^2^ values in Table 6, the FWO method was superior to other methods regarding the analysis of pure MEA and MEA mixtures and is widely used in thermokinetics analysis^32^.

**Table 6:** Results of *E*_a_ and *R*^2^ calculations obtained using various nonisothermal kinetic methods.

Samples		ASTM E698		KAS		Starink		FWO	
		*E*_a_ (kJ/mol)	*R* ^2^	*E*_a_ (kJ/mol)	*R* ^2^	*E*_a_ (kJ/mol)	*R* ^2^	*E*_a_ (kJ/mol)	*R* ^2^
MEA		26.2	0.988	26.2	0.984	26.7	0.985	31.2	0.989
MEA + CuBr_2_	1st Peak	35.5	0.939	79.4	0.777	79.8	0.781	81.6	0.806
	2nd Peak	116.9	0.990	124.4	0.992	124.9	0.992	127.4	0.993
MEA + ZnBr_2_	1st Peak	41.5	0.818	45.1	0.964	45.4	0.964	48.5	0.971
	2nd Peak	117.9	0.988	111.7	0.994	112.3	0.994	115.4	0.995

## Conclusions

In this study, the thermal decomposition and nonisothermal kinetics of pure MEA and MEA mixed with copper (II) and zinc (II) were illustrated using TG and DSC. In the TG tests, the thermal decomposition of MEA mixed with CuBr_2_ and ZnBr_2_ began at 75.2 and 60.3 °C, respectively; all mixtures produced prior decomposition reactions compared with pure MEA (89.7 °C).

In the DSC analysis, two exothermic peaks were observed after the addition of CuBr_2_ and ZnBr_2_ to MEA. Although no notable change in Δ*H*_d_ occurred, *T*_0_ decomposed early at 79.2 and 69.6 °C in the copper and zinc mixtures, respectively. These results suggested that metal ions may provoke early reactions during the manufacturing process. These advanced reactions may lead to thermal decomposition of the material of interest at low temperatures, resulting in thermal hazards. Moreover, nonisothermal methods, namely the ASTM E698, KAS, Starink, and FWO methods, were used to analyze the decomposition kinetics of MEA and MEA mixtures. The fitting was insufficient in a single conversion compared with the *R*^2^ in different conversions. The *E*_a_ of pure MEA was 28.7 ± 2.5 kJ/mol. The *E*_a_ results of the copper and zinc mixtures were 80.5 ± 1.1 and 46.8 ± 1.7 kJ/mol, respectively. Chemical reactions caused by the addition of metal ions resulted in these results of higher *E*_a_ for the mixtures.

Ethanolamine is widely used in petrochemical industries, and the operation of such enterprises involves numerous unsafe processes. Because of the advanced reactions of metal ions to ethanolamine, corroded pipelines and equipment should be promptly replaced to reduce the likelihood of chemical disasters and ensure plant safety.
